# Organic Membrane Fouling in Advanced Water Purification: Mechanisms, Bulk-Phase Aggregation, and Control Strategies

**DOI:** 10.3390/membranes16080271

**Published:** 2026-08-14

**Authors:** Guoqing Wang, Bihui Niu, Geng Tang, Tianxiang Wang, Ningqing Lv

**Affiliations:** 1 School of Energy and Chemical Engineering, Puyang Vocational and Technical College, Puyang 457001, China; 2 Shanghai Energy Construction Engineering Design Research Co., Ltd., Shanghai 200135, China; 3 State Key Laboratory of Environmental Criteria and Risk Assessment, Chinese Research Academy of Environmental Sciences, Beijing 100012, China

**Keywords:** membrane fouling, organic aggregates, metal ions, advanced purification, emerging contaminants, fouling control

## Abstract

Membrane separation has become a key technology for advanced water purification and control of emerging contaminants because of its high separation efficiency, low chemical demand, and ease of integration. However, organic membrane fouling induced by the coupling of dissolved organic matter and coexisting metal ions remains a major obstacle to stable and efficient membrane operation. This review focuses on metal ion-mediated formation of organic aggregates in the bulk solution and their governing role in membrane fouling behavior. The review summarizes how metal ions regulate organic aggregate formation through distinct dominant mechanisms. Na^+^ mainly screens electrostatic repulsion, Ca^2+^ promotes ion bridging and cross-linking, and Mg^2+^ often induces weaker bridging or hydration-mediated effects due to its stable hydration shell. The review further discusses the dual effects of mixed foulants and dynamic fouling layers on the rejection of emerging contaminants. In addition, current control strategies, including pre-coagulation, pre-oxidation, and catalytic functional membranes, are evaluated from the perspective of regulating aggregate structures and interrupting interfacial deposition. Finally, future research should shift from membrane-interface-centered analysis to bulk-phase aggregation, establish quantitative structure–effect relationships between aggregate properties and fouling behavior, and promote fouling-control strategies from mechanistic effectiveness toward engineering practicality.

## 1. Introduction

Membrane fouling is one of the most critical bottlenecks limiting the long-term stability of membrane-based water treatment systems. Over the past few decades, extensive efforts have been devoted to elucidating fouling mechanisms and developing mitigation strategies, leading to a conventional research framework centered on flux decline monitoring, resistance analysis, and fouling-layer characterization [1]. Within this framework, membrane fouling is largely understood as foulant deposition on membrane surfaces or within membrane pores, and research has therefore focused mainly on the composition, structure, and reversibility of interfacial fouling layers [2]. However, growing evidence suggests that organic membrane fouling is not determined solely by interfacial deposition [3,4]. In natural waters and municipal wastewaters, major organic foulants such as polysaccharides, proteins, and humic substances often undergo conformational rearrangement and heterogeneous aggregation before reaching the membrane interface, especially in the presence of coexisting ions such as Na^+^, Ca^2+^, and Mg^2+^ [5,6,7]. The resulting aggregates, characterized by altered size, structure, and surface charge, act as the actual fouling units that govern subsequent deposition behavior and fouling-layer formation [8,9,10].

Although many studies have reported the fouling behavior of single or mixed organic systems under different water chemistry conditions, the formation kinetics, structural evolution, and environmental responsiveness of organic matter–metal ion aggregates remain insufficiently understood [4,11]. Ca^2+^ and Mg^2+^ can induce markedly different, or even opposite, aggregation behaviors in the same polysaccharide system [12], while ionic strength and pH can substantially alter humic fouling behavior [13]. However, quantitative relationships between aggregate properties, such as formation rate, crosslinking density, and hydration degree, and fouling-layer properties, such as permeability, compactness, and reversibility, have not yet been established.

Membrane fouling also affects separation performance beyond flux decline. The dynamically accumulated fouling layer can act as a secondary interface with adsorption and sieving functions, altering the transport and rejection of trace organic micropollutants depending on both fouling-layer properties and pollutant characteristics [14,15]. Therefore, fouling control is essential for maintaining membrane productivity and ensuring effluent quality.

From this perspective, this review aims to reorganize organic membrane fouling from the viewpoint of metal ion-mediated bulk-phase aggregation rather than providing a general inventory of membrane-fouling phenomena. Particular attention is given to how metal-ion–organic-foulant interactions in the bulk solution regulate aggregate properties and how these bulk-phase properties subsequently govern interfacial deposition, emerging-contaminant rejection, inorganic scaling, and fouling-layer reversibility. Compared with previous reviews that mainly emphasize membrane-surface modification, fouling-layer characterization, or operational mitigation, this review identifies upstream aggregate formation as a key missing link between feed-water chemistry and downstream membrane performance. By connecting aggregate evolution with model-based characterization and control strategies such as pre-coagulation, pre-oxidation, and catalytic functional membranes, this review is expected to support more predictive and transferable strategies for membrane-fouling control in advanced water treatment.

## 2. Current Understanding of Membrane Fouling

### 2.1. Mechanistic Insights into Organic Foulant-Induced Membrane Fouling

**Figure 1:**
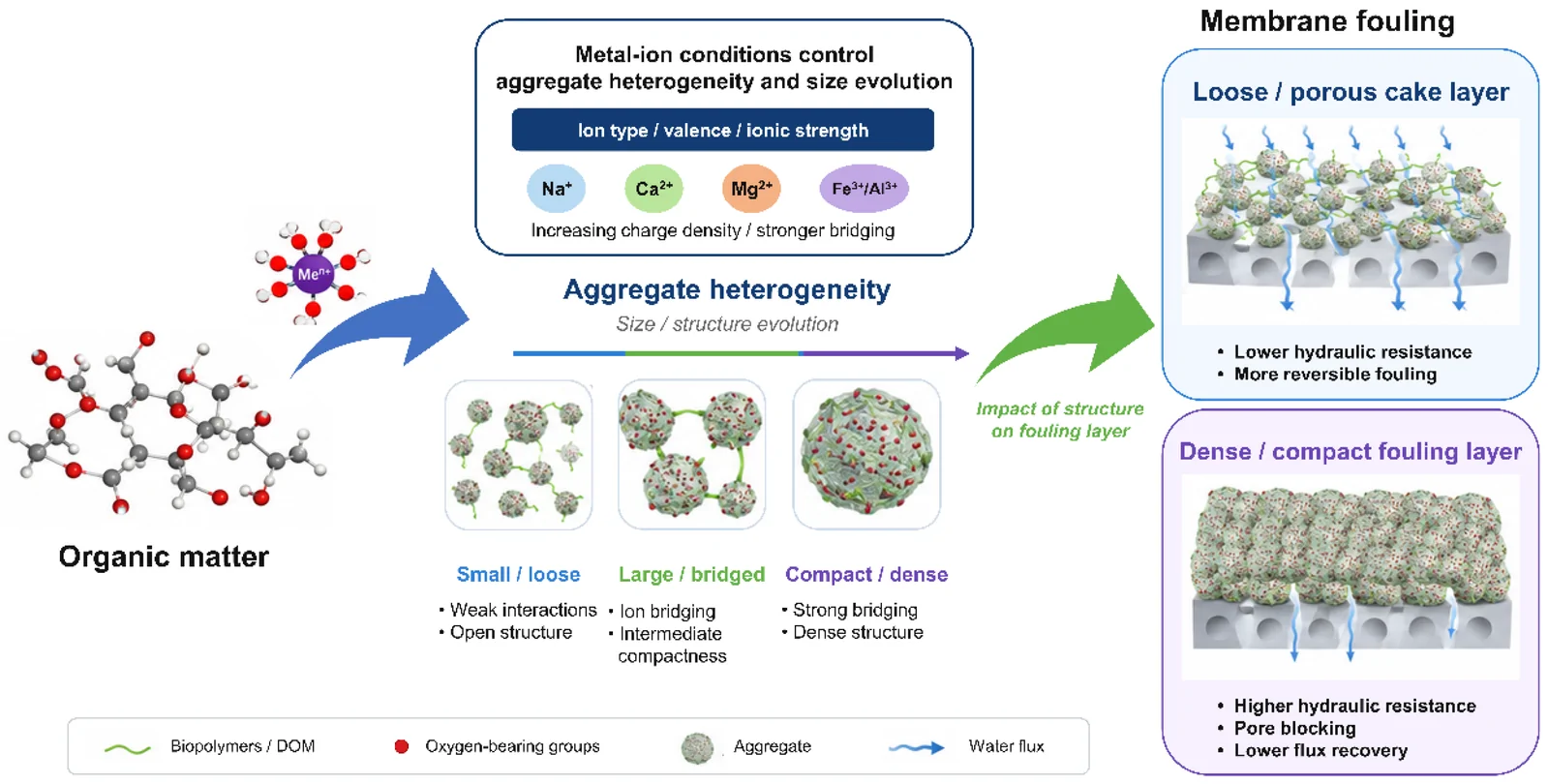
Schematic illustration of organic membrane fouling.

Macromolecular organic foulants, especially polysaccharides, proteins, and humic substances, contain abundant functional groups, exhibit flexible molecular structures, and show strong interfacial activity, making them prone to accumulation on membrane surfaces and within membrane pores. Under realistic operating conditions, these organics usually coexist with inorganic ions such as Na^+^, Ca^2+^, and Mg^2+^, so membrane fouling essentially evolves into a coupled organic–inorganic deposition process [6]. Inorganic ions interact with organics through ion bridging, electrostatic interactions, and hydration-mediated associations, generating aggregates whose structures and deposition behaviors determine fouling-layer compactness, reversibility, and hydraulic resistance (Figure 1) [8,16]. These ion-mediated interactions are therefore central to understanding organic membrane fouling and developing targeted fouling-control strategies.

#### 2.1.1. Humic Substance Fouling

Humic substances are among the most representative organic components in natural waters and account for a major fraction of total natural organic carbon. They are generally regarded as supramolecular assemblies formed by low-molecular-weight biomolecules through weak intermolecular interactions, and their physicochemical state is highly sensitive to environmental conditions [17]. Under high concentration (>3.5 g/L), low pH (<3.5), and high ionic strength (>50 mM), humic substances tend to exist as rigid, electrically neutral colloidal particles, whereas under dilute, alkaline, and low-ionic-strength conditions they behave more like flexible linear polyelectrolytes [18]. During membrane filtration, the abundant carboxyl and phenolic groups on humic molecules can interact with metal ions through double-layer compression, charge neutralization, ion bridging, and hydration effects, thereby forming heterogeneous aggregates that deposit on the membrane surface [19,20]. Previous studies have shown that K^+^ and Ca^2+^ exert markedly different influences on humic-fouling behavior [20]. Ca^2+^ tends to promote chain extension through ion bridging, leading to relatively reversible fouling layers, whereas K^+^ mainly induces molecular compression through electrostatic screening, resulting in more irreversible fouling. In addition, the distinct hydration structures of K^+^ and Ca^2+^ further affect fouling-layer architecture and humic rejection behavior. Solution pH and ionic strength can also change the interaction mode between humic substances and metal ions, thereby altering fouling-layer structure [21]. At high ionic strength, both Ca^2+^ and Mg^2+^ may alleviate humic fouling, although by different pathways. Ca^2+^ promotes humic aggregation and restructures the fouling layer, whereas Mg^2+^ suppresses aggregate deposition through strong hydration repulsion. Owing to its stronger binding with humic functional groups, Ca^2+^ often shows a more pronounced mitigation effect than Mg^2+^ under comparable conditions [19,22]. However, this Ca^2+^-dominant mitigation effect should not be generalized across all membrane types. Membrane pore size, surface charge, hydrophobicity, roughness, and salt-rejection ability can influence whether Ca^2+^-induced humic aggregation reduces pore blocking or instead enhances interfacial deposition and concentration polarization. These ion-dependent effects may also vary with hydrodynamic configuration. Under dead-end or weak-shear conditions, concentration polarization can enhance the local enrichment of humic substances and salts near the membrane surface, favoring aggregate deposition or compact fouling-layer formation. In contrast, cross-flow operation promotes shear-induced back transport and can partially weaken surface accumulation. Therefore, the apparent roles of Ca^2+^ and Mg^2+^ should be interpreted together with membrane surface properties, bulk aggregation behavior, and interfacial transport conditions.

#### 2.1.2. Protein Fouling

Proteins are major constituents of soluble microbial products and extracellular polymeric substances and are therefore common membrane foulants [23]. Bovine serum albumin (BSA) is frequently used as a model protein in fouling studies. In single-BSA systems, fouling mainly arises from the attachment of BSA molecules to the membrane surface and to already deposited protein layers [24]. When metal ions coexist, BSA-ion interactions can alter protein charge screening, hydration state, intermolecular association, and foulant–membrane interactions, thereby changing aggregate formation and subsequent fouling behavior [25]. Under high-ionic-strength conditions, with NaCl often used as a background electrolyte, BSA fouling may remain relatively mild at the early stage of filtration, but can become more severe as filtration proceeds. This late-stage increase in fouling can be attributed primarily to the increase in total ionic strength caused by salt accumulation near the membrane surface, rather than to a Na^+^-specific effect. The elevated ionic strength promotes charge screening, protein dehydration, and salting-out, thereby weakening hydration repulsion and facilitating BSA deposition [26]. The contrasting effects of Ca^2+^ and Mg^2+^ reported in different BSA fouling studies can be largely explained by differences in concentration polarization. Under dead-end or weak-shear conditions, local accumulation of BSA and salts can favor the formation of loose cake layers, whereas under cross-flow conditions, shear-induced back transport reduces bulk accumulation, but locally enriched Ca^2+^ or Mg^2+^ can still aggravate BSA deposition through charge screening and stronger foulant–membrane interactions [27,28].

#### 2.1.3. Polysaccharide Fouling

Compared with proteins and humic substances, polysaccharides generally exhibit a higher fouling propensity owing to their structural diversity, high molecular weight, and strong gel-forming characteristics [29,30]. Recent studies have shown that polysaccharide fouling is closely associated with the formation of transparent exopolymer particles (TEP), which are gel-like acidic polysaccharide aggregates with three-dimensional network structures and are now recognized as active foulants in membrane filtration processes [8,31,32]. Ion bridging is a dominant mechanism governing TEP-associated fouling, but its effect strongly depends on both polysaccharide structure and ion type [8,29]. For linear acidic polysaccharides such as sodium alginate and guar gum, Ca^2+^ can effectively promote intermolecular crosslinking through charge neutralization and bridging interactions, thereby enhancing aggregate formation and aggravating membrane fouling [8,29]. By contrast, Mg^2+^ usually exhibits weaker bridging capability because its strongly bound hydration shell limits direct coordination with polysaccharide functional groups, leading instead to looser aggregate structures and less compact fouling layers [8]. This Ca^2+^/Mg^2+^ contrast indicates that polysaccharide fouling is controlled not only by ion valence but also by ion-specific hydration and coordination chemistry. Ca^2+^ can more readily interact with carboxyl groups and form intermolecular bridges, thereby increasing cross-linking density, aggregate size, and gel-layer compactness. In contrast, Mg^2+^ has a stronger hydration shell, which hinders direct coordination with polysaccharide chains and may preserve more hydrated and deformable aggregate structures. Therefore, even within the same divalent-cation category, Ca^2+^ and Mg^2+^ can induce markedly different aggregate architectures and fouling-layer permeability. This mechanism explains why the same polysaccharide system may show severe Ca^2+^-enhanced fouling but weaker or even alleviated fouling in the presence of Mg^2+^.

Monovalent ions can also influence polysaccharide fouling, although their effects are generally weaker than those of divalent ions. Na^+^ and K^+^ both facilitate polysaccharide deposition through electrostatic screening, but Na^+^ tends to induce more severe fouling by strengthening intermolecular attraction and promoting continuous foulant accumulation, whereas K^+^ more often leads to relatively loose and reversible deposits [33]. In addition to regulating aggregate formation in the bulk phase, metal ions can also alter foulant–membrane interactions. Ca^2+^ may shift polysaccharide–membrane interactions from repulsive to attractive, thereby accelerating interfacial deposition and promoting the formation of dense fouling layers with low permeability [8,29].

Other metal ions can further diversify polysaccharide fouling behavior. Mn^2+^ has been reported to enlarge TEP size and reduce adhesion/cohesion energy barriers through complexation with polysaccharide carboxyl groups, thereby aggravating membrane deposition [34]. High-valence ions such as Al^3+^ and Fe^3+^ can also participate in TEP formation, although their preferred coordination modes and binding sites differ from those of Ca^2+^, resulting in distinct aggregate structures and fouling-layer properties [35]. Overall, polysaccharide fouling is governed by both polymer properties and ion-mediated aggregation in the bulk solution, with aggregate structure ultimately determining the permeability, reversibility, and mechanical stability of the fouling layer [8,34,35]. Hydrodynamic configuration is also important for interpreting polysaccharide fouling. In dead-end or low-shear filtration, Ca^2+^-bridged polysaccharide aggregates and gel-like networks are more readily accumulated at the membrane surface, intensifying cake compression and hydraulic resistance. Under cross-flow conditions, shear-induced back transport can reduce surface accumulation, but dense or highly adhesive Ca^2+^-polysaccharide aggregates may still deposit when local concentration polarization remains strong. Therefore, the same ion-mediated aggregation mechanism may lead to different fouling outcomes depending on the balance between bulk aggregate formation, surface transport, and shear removal.

#### 2.1.4. Combined Organic Fouling

**Figure 2:**
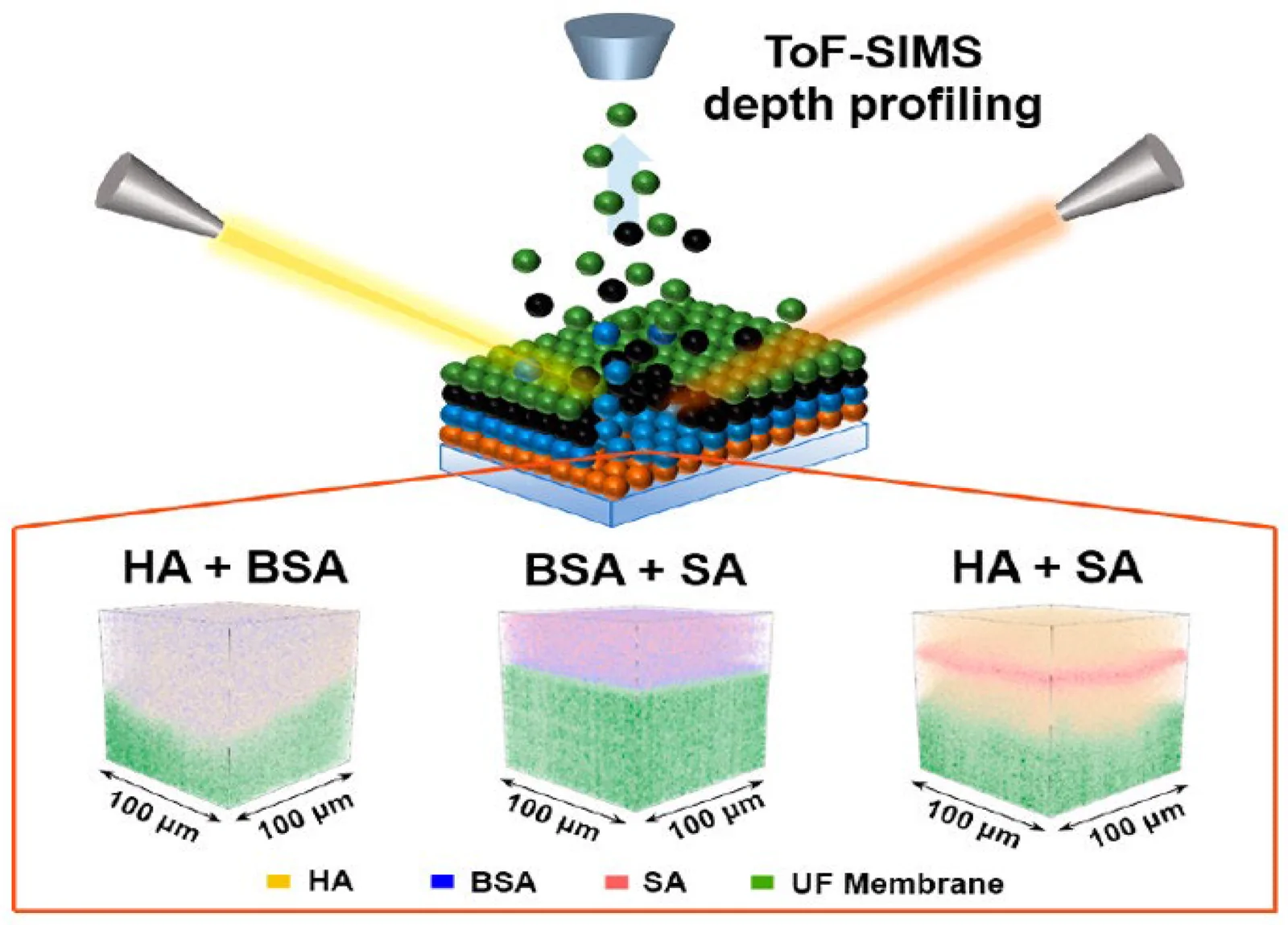
Composite organic fouling. Reprinted with permission from [3]. Copyright 2021 American Chemical Society.

In practical wastewater treatment, feed streams contain multiple interacting foulants, so membrane fouling often reflects non-additive and intensified effects rather than the simple deposition of individual components (Figure 2) [36,37,38]. In humic substance-protein systems, humic molecules can interact with proteins through carboxyl and amide groups, forming encapsulation structures that suppress protein adsorption on membrane surfaces [39]. In polysaccharide–protein systems, large non-covalent aggregates are often formed through electrostatic interactions, resulting in porous and weakly cohesive fouling layers. In polysaccharide–humic systems, the two components may deposit asynchronously and form sandwich-like structures with synergistic fouling effects [3]. Coexisting metal ions further modulate mixed fouling. For example, Ca^2+^ can increase fouling resistance in polysaccharide–protein mixtures and mediate interactions between polysaccharides and humic substances, making the fouling behavior more similar to polysaccharide-dominated systems [40,41].

Taken together, existing studies have shown that ion type, ionic strength, ion valence, pH, and foulant properties strongly influence flux decline, fouling-layer structure, and the dominant fouling mechanism. However, research has largely focused on interfacial deposition and its final manifestations, whereas the formation and evolution of organic matter–metal ion aggregates in the bulk solution remain insufficiently understood. This disconnect between upstream aggregation mechanisms and downstream fouling behavior limits predictive capability and precise control of membrane fouling. Therefore, future research should shift toward the bulk phase and focus on aggregate formation, structural heterogeneity, and dynamic evolution, which are essential for developing mechanistic models and rational fouling-control strategies.

Table 1 summarizes representative quantitative relationships between ionic conditions, aggregate properties, and membrane-fouling performance. Collectively, these studies demonstrate that membrane fouling is governed not only by ion concentration and valence, but also by ion-specific hydration, organic-matter structure, ionic strength, and ion-addition mode. However, direct and systematically comparable relationships between bulk aggregate properties and downstream fouling-layer characteristics remain limited. Future studies should combine bulk-phase aggregate characterization with membrane interface analysis under controlled ionic conditions. Aggregate size distribution, surface charge, and aggregation kinetics can be quantified using dynamic light scattering, laser diffraction, zeta-potential analysis, and time-resolved turbidity measurements. Ion-binding and functional-group interactions can be further examined by fluorescence spectroscopy, FTIR, XPS, and isothermal titration calorimetry. These aggregate descriptors should be coupled with filtration indicators, including flux decline, hydraulic resistance, flux recovery, QCM-D response, AFM interaction forces, and SEM/CLSM-based fouling-layer morphology. Such paired measurements would help establish quantitative relationships between aggregate size, charge, compactness, and fouling-layer permeability, reversibility, and hydraulic resistance.

#### 2.1.5. Organic Foulant-Mediated Inorganic Scaling

In addition to the effects of inorganic ions on organic fouling, organic foulants can also regulate inorganic scaling. In pressure-driven membrane processes, organic deposition often occurs before mineral precipitation. The pre-existing organic layer can change membrane surface charge, roughness, hydrophilicity, local ion enrichment, and nucleation sites, thereby promoting or suppressing the formation of gypsum, silica, and calcium carbonate scales [11,49,50].

Gypsum scaling is a typical example. Organic-fouled NF membranes were reported to suffer more severe gypsum-induced flux decline than virgin membranes, with the scaling tendency following the order of virgin membrane < BSA-fouled membrane < HA-fouled membrane < SA-fouled membrane [49]. This behavior was mainly attributed to the Ca^2+^-binding capacity of carboxyl-rich organic foulants. HA and SA can enrich Ca^2+^, promote heterogeneous nucleation, and accelerate surface crystallization, while BSA shows a weaker scaling-promoting effect [49]. These results indicate that the chemical nature of the pre-existing organic layer, especially carboxyl density and Ca^2+^ affinity, strongly determines subsequent gypsum scaling [11].

For silica scaling, the mechanism is different because silica deposition is governed mainly by silica polymerization rather than simple salt crystallization [50]. RO membranes preconditioned by HA, SA, or BSA showed enhanced silica scaling because the organic fouling layer provided interfacial space and active sites for silica deposition. High pH and Ca-rich conditions favored dense and irreversible silica scaling layers, whereas Mg-rich conditions tended to generate looser deposits with lower hydraulic resistance [50]. Once silica scaling occurred, the organic fouling layer became much less reversible, suggesting that organic-silica coupling can transform relatively mild organic fouling into persistent composite fouling [50].

Therefore, organic matter has a dual role in inorganic scaling. Dissolved organic matter in the bulk phase may inhibit crystal growth by complexing Ca^2+^ or blocking crystal growth sites, whereas deposited organic layers may promote scaling through local ion enrichment, heterogeneous nucleation, and cake-enhanced concentration polarization [11,49,51]. Future studies should distinguish bulk-phase inhibition from surface-induced scaling and combine filtration tests with interfacial characterization techniques such as QCM-D, AFM, X-ray reflectivity, grazing-incidence X-ray fluorescence, SEM-EDS, and mineralogical analyses [49,51].

### 2.2. Model-Based Characterization of Membrane Fouling and Its Applicability

The evolution of membrane fouling cannot be understood solely through experimental observation; mathematical models are also required to identify fouling mechanisms and quantitatively describe fouling dynamics. Existing fouling models can generally be divided into mechanistic models and data-driven models. The former include the resistance-in-series model, XDLVO theory, and the Hermia model, which interpret fouling in terms of resistance partitioning, interfacial interaction energy, and pore-blocking behavior. The latter use data-driven approaches to predict fouling evolution from multi-source operational and water-quality data [52].

The resistance-in-series model is based on the additive contributions of intrinsic membrane resistance, pore resistance, and cake-layer resistance and is widely used to explain membrane fouling. XDLVO theory focuses on the microscopic scale by quantifying van der Waals, electrostatic, and polar interactions between foulants and between foulants and the membrane surface [29]. The Hermia model describes fouling through blocking kinetics and classifies fouling into four classical mechanisms: complete blocking, standard blocking, intermediate blocking, and cake filtration, as exhibited in Table 2 [53].

**Table 2:** Classical fouling mechanisms described by the Hermia model [53].

Fouling Mechanism	Mathematical Formula	Characteristics	Schematic Diagram
Complete blocking model	$\frac{J}{J_{0}}=e^{-K_{a}J_{0}t}$	Complete blockage and coverage of membrane pores by foulants	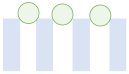
Standard blocking model	$\frac{J}{J_{0}}=(1+K_{s}J_{0}t{)}^{-2}$	Pore constriction due to deposition inside membrane pores	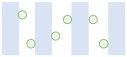
Intermediate blocking model	$\frac{J}{J_{0}}=(1+K_{a}J_{0}t{)}^{-1}$	Accumulation of foulants leading to partial pore blocking	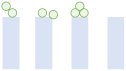
Cake filtration model	$\frac{J}{J_{0}}={\left(1+2K_{c}R_{r}J_{0}t\right)}^{-0.5}$	Formation of a multilayer fouling cake on the membrane surface	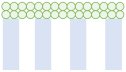

Light‑blue bars: membrane and pores; green‑circled circles: foulant particles. Four panels show four typical Hermia membrane fouling mechanisms with different foulant deposition patterns.

Compared with mechanistic models, artificial intelligence provides a data-driven route for predicting complex membrane-fouling behavior. Representative algorithms, including artificial neural networks, support vector machines, fuzzy logic, genetic programming, and other machine-learning models, have been used to predict flux decline, transmembrane pressure evolution, fouling rate, and overall membrane performance by integrating operational and water-quality variables such as pressure, flux, foulant concentration, ionic strength, pH, and temperature [52,54,55]. Their ability to capture nonlinear relationships makes them useful for fouling early warning, process optimization, and operational control.

However, AI models depend strongly on data quality, sample size, variable selection, and the representativeness of training datasets [52,54,55]. Their limited physical interpretability also creates a “black-box” problem, making them more suitable as predictive and decision-support tools than as replacements for mechanistic models.

## 3. Impacts of Membrane Fouling on the Removal of Emerging Contaminants

In practical membrane treatment, organic fouling can substantially alter the removal of emerging contaminants. Polysaccharides, proteins, and humic substances dynamically accumulate on membrane surfaces, changing surface charge, hydrophobicity, pore characteristics, and the local membrane-fouling-layer microenvironment, thereby affecting contaminant transport, rejection, and long-term separation stability [56,57].

Available studies indicate that the influence of membrane fouling on emerging-contaminant rejection depends strongly on contaminant properties [56]. For negatively charged antibiotics, humic fouling can substantially reduce rejection by nanofiltration membranes, and the coexistence of Ca^2+^ may further decrease removal efficiency [56]. For neutral and hydrophobic estrogens, the balance among size exclusion, hydrophobic interactions, and concentration polarization can also lead to lower rejection after fouling [56]. Furthermore, cake-enhanced concentration polarization may suppress the removal of nonionic pharmaceuticals and personal care products such as acetaminophen, carbamazepine, and bisphenol A [57,58].

By contrast, membrane fouling may also enhance contaminant rejection under specific conditions. Humic-acid/Ca^2+^ fouling was found to produce different rejection responses for acetaminophen, sulfamethoxazole, and triclosan [59]. These differences resulted from the combined effects of size exclusion, electrostatic interactions, and cake-enhanced concentration polarization, with their relative contributions depending on contaminant properties and membrane characteristics. Thus, the fouling layer should be regarded as a dynamic secondary separation interface rather than merely an additional hydraulic resistance. In electroactive membrane systems, different organic foulants can exert opposite effects. Alginate fouling may enhance sulfamethoxazole degradation by adsorbing the pollutant and facilitating its transfer from the bulk solution to the reactive membrane surface, whereas humic-acid fouling may inhibit degradation [60].

Collectively, the studies summarized in Table 3 demonstrate that fouling layers act as dynamic secondary separation interfaces. They may enhance contaminant removal through adsorption, electrostatic exclusion, and additional size sieving, but may also reduce rejection or degradation by intensifying concentration polarization, limiting mass transfer, or altering access to reactive membrane surfaces. Future membrane-process design should therefore consider foulant composition, contaminant polarity and molecular size, membrane properties, and water-matrix complexity together. Quantitative relationships between fouling-layer microstructure and contaminant-removal behavior are particularly needed to support more precise process optimization.

## 4. Integrated Strategies for Simultaneous Water Purification and Fouling Control

Both the purification performance and fouling behavior of membrane systems are governed by feed-water chemistry, membrane properties, and operating conditions. Adjusting operating parameters or modifying membrane surfaces can improve flux and suppress foulant deposition, but these approaches generally delay rather than eliminate fouling [61,62]. More effective control may require either upstream intervention before foulants reach the membrane or in situ treatment at the membrane interface. Pretreatment technologies such as adsorption, coagulation, flocculation, and oxidation can remove, aggregate, or transform foulants upstream, thereby reducing the fouling load imposed on the membrane surface [63,64]. In parallel, functional membranes with antifouling or self-cleaning capabilities provide a direct route for suppressing foulant deposition or degrading deposited foulants at the membrane interface [65]. This section discusses pre-coagulation, pre-oxidation, and functional membrane integration as representative strategies for dual-purpose purification and fouling control.

### 4.1. Fouling Mitigation Through Pre-Coagulation

Coagulation pretreatment destabilizes hydrophobic substances and macromolecular organics through charge neutralization, bridging, entrapment, and sweep flocculation, forming larger flocs that are more readily retained on the membrane surface. This process improves fouling-layer structure, enhances particle back-transport, and reduces both reversible and irreversible fouling [66]. Coagulant selection strongly affects process performance. For example, high-dose polyaluminum chloride can induce specific coordination between Al^3+^ and carrageenan, shifting the fouling layer from a gel-like structure toward a cake layer and thereby mitigating fouling [67].

Nevertheless, single-coagulant systems often suffer from high dosage requirements, limited removal efficiency for some pollutants, and excessive sludge production. Dual-coagulant systems combining metal salts with organic coagulant aids can improve water quality while strengthening fouling control [68]. In coagulation/ultrafiltration treatment of river water, cationic lignin-based flocculants combined with polymeric aluminum coagulants have been shown to shift fouling from intermediate blocking toward cake filtration and reduce both external and internal reversible resistances, leading to a substantial drop in total resistance [68].

Coagulation is less effective for low-molecular-weight hydrophilic organics, which can still enter membrane pores and cause irreversible fouling. To address this limitation, micro/nanobubble-assisted coagulation has been proposed and can significantly lower irreversible fouling and transmembrane pressure [63]. This improvement has been attributed to enhanced removal of hydrophilic organics, increased floc compactness, and reconstruction of floc-formation pathways, which together improve floc shear resistance and regeneration capability [63]. Hybrid oxidation–coagulation pretreatment can further improve fouling control. For example, trace homogeneous Fenton pretreatment combines organic-matter oxidation with Fe-mediated coagulation [69]. Oxidation alters the charge characteristics and hydroxyl-group abundance of organic matter, strengthening electrostatic repulsion and weakening hydrogen bonding with the membrane surface, whereas coagulation promotes the formation of larger flocs. Together, these processes produce more porous and permeable fouling layers and substantially improve flux recovery [69].

### 4.2. Fouling Mitigation Through Pre-Oxidation

Pre-oxidation mitigates fouling at the source by generating radical and non-radical oxidizing species in situ, which degrade macromolecular organics into smaller, less harmful compounds or even mineralize them [64,70,71]. Pretreatment with magnetic biochar beads combined with peroxymonosulfate activation before ultrafiltration can remove UV-absorbing organics and dissolved organic carbon, reduce intermediate blocking and cake filtration, and lower total and reversible fouling resistance in practical wastewater treatment [71].

Nanoconfined carbon-based catalysts can further enhance pretreatment performance by increasing the local concentration of reactive species and thereby improving the degradation efficiency of organic foulants [64]. Carbon nanotubes encapsulating cobalt nanoparticles can activate peroxymonosulfate and degrade bisphenol A and representative foulants, including polysaccharides, proteins, and humic substances. They can also reduce aromatic, fluorescent, and medium-to-high-molecular-weight organic fractions [64]. These effects can markedly lower both reversible and irreversible fouling resistance and delay the formation of dense fouling layers [64].

In practical wastewater treatment, pre-oxidation must also account for nutrient removal. A series of peracetic acid-based pretreatment processes, including Fe(VI)/peracetic acid and Fe(II)/peracetic acid systems, has therefore been developed [70]. Among these processes, Fe(II)-activated peracetic acid showed the strongest fouling-control performance under the reported experimental conditions [70]. It reduced total fouling resistance, removed fluorescent and hydrophobic organic fractions, weakened foulant–membrane attraction, and simultaneously enhanced phosphorus removal and emerging-contaminant degradation. Non-radical oxidation pathways such as singlet oxygen have also attracted attention because of their longer-lived reactive species and selective oxidation behavior toward electron-rich organics and pathogens [72]. However, in some comparative studies, non-radical pathways have shown lower pollutant-removal performance than sulfate-radical pathways and produced smaller increases in the interaction-energy barriers between foulants and membranes [73].

Despite their effectiveness, pre-coagulation and pre-oxidation must be evaluated against the additional process complexity, cost, and energy demand introduced by pretreatment units. Moreover, most of these technologies remain at the laboratory stage, and practical demonstrations for emerging-contaminant control and membrane-fouling mitigation in real wastewater treatment plants are still limited. Future evaluation should therefore shift from asking whether these strategies are effective to whether they are practical and scalable.

Future research should focus on translating these pretreatment strategies from proof-of-concept studies to process-compatible modules. Oxidation or coagulation intensity should be optimized based on the minimum effective dose rather than the maximum degradation efficiency, because excessive chemical input may increase cost, residual oxidants, sludge production, by-product formation, and downstream membrane damage. Long-term continuous-flow experiments using real wastewater matrices are also needed to evaluate flux stability, contaminant removal, oxidant consumption, catalyst leaching, membrane-cleaning frequency, and fouling reversibility. In addition, energy and chemical consumption should be reported together with conventional performance indicators to support techno-economic and life-cycle assessments. Low-dose hybrid processes, such as mild coagulation–oxidation, selective non-radical oxidation, and catalytic pretreatment coupled with membrane filtration, should be prioritized to balance fouling control, emerging-contaminant removal, operational simplicity, and engineering scalability.

### 4.3. Functional Membranes for Synergistic Purification and Fouling Control

To avoid the additional cost and energy demand of upstream pretreatment, integrated membranes combining separation and catalytic oxidation have been developed for simultaneous water purification and fouling control [65]. These catalytic membranes enable in situ degradation of foulants and emerging contaminants at the membrane interface and have been coupled with persulfate oxidation, Fenton oxidation, and photocatalysis [65].

Integrated coagulation/co-catalytic membrane systems combine upstream foulant removal with interfacial pollutant degradation [74]. In a representative Fe^3+^-MoS_2_ system, Fe^3+^ acted both as a coagulant for dissolved organic matter and as a co-catalyst with membrane-embedded MoS_2_ nanosheets for peroxymonosulfate activation. This integration enhanced pollutant degradation while reducing the organic load reaching the catalytic membrane surface [74]. Other studies have designed oxygen-vacancy-mediated nanoarray electro-Fenton membranes to investigate how catalytic-layer structure influences antibiotic degradation and fouling mitigation [75]. Under hydroxyl-radical oxidation, such membranes can achieve very high degradation efficiency and excellent flux recovery with only slight irreversible fouling [75].

Single-atom catalysts have emerged as particularly promising membrane-embedded catalysts because of their high atomic efficiency, uniform dispersion in membrane layers, and minimal disruption to membrane macrostructure and separation performance [76]. The single-atom Fe loaded onto photocatalytically active g-C_3_N_4_ has been used to construct catalytic membranes capable of activating peracetic acid for the degradation of acetaminophen and macromolecular organics while maintaining membrane permeability and structural stability [77]. In these systems, photogenerated charge carriers and in situ formed oxidizing species cooperate to improve pollutant degradation, while spatial confinement at the catalytic membrane surface suppresses the shielding of active sites by macromolecular foulants [77].

Catalytic membranes can improve flux recovery and reduce foulant accumulation through interfacial pollutant degradation, but their long-term stability requires careful evaluation. In radical-based systems, reactive species such as ^•^OH and SO_4_^•-^ may also oxidize polymeric membrane matrices, including PES and PVDF, leading to chain scission, pore-structure changes, loss of selectivity, or catalyst detachment. Therefore, stable catalytic membranes require protective architectures that localize reactive species within the catalytic surface layer, reduce direct exposure of the polymer backbone, and minimize catalyst leaching. Representative designs include inorganic or carbonaceous catalytic coatings, hydrophilic nanoarray layers, nanosheet-anchored catalysts, and single-atom coordination structures. Existing studies have provided short-term evidence of stability through repeated-cycle testing, flux-recovery measurements, post-reaction structural characterization, and catalyst-leaching analyses. However, continuous long-term operation under realistic water matrices remains insufficiently demonstrated. Therefore, catalytic membranes should be described as promising for improving operational durability and reducing cleaning frequency, rather than being assumed to extend membrane lifetime.

As shown in Table 4, pretreatment strategies can substantially reduce fouling and improve flux recovery, whereas integrated catalytic membranes provide additional pollutant-degradation functions. However, the reported studies differ in feed-water composition, membrane configuration, operating conditions, and performance indicators. Standardized evaluation using flux stability, fouling resistance, cleaning recovery, contaminant removal, chemical consumption, and catalyst stability is therefore needed to support meaningful comparison and engineering selection.

## 5. Conclusions and Perspectives

Organic membrane fouling remains a major barrier to stable membrane-based water treatment. In the presence of coexisting metal ions, polysaccharides, proteins, humic substances, and their mixtures form aggregates through ion bridging, electrostatic screening, and hydration effects. These aggregates drive interfacial deposition, pore blockage, and fouling-layer evolution, while also reshaping the membrane microenvironment and altering the transport and rejection of emerging contaminants.

For process characterization, Hermia-type models remain useful for identifying macroscopic fouling mechanisms, while artificial intelligence can improve predictive performance under complex conditions. However, mechanistic models often simplify organic foulant–ion-membrane interactions, whereas AI models remain limited by data dependence and poor interpretability. Membrane fouling also exerts a dual effect on advanced purification, enhancing contaminant rejection through secondary sieving and adsorption but reducing removal through intensified concentration polarization.

Pre-coagulation, pre-oxidation, and functional membrane integration offer promising routes for simultaneous water purification and fouling control. However, their practical application remains constrained by cost, energy demand, long-term stability, and scale-up challenges, and direct comparison remains difficult because current studies use inconsistent operating conditions and performance indicators. Overall, research on organic membrane fouling is progressing from phenomenological description toward mechanistic analysis and from single-purpose fouling mitigation toward the simultaneous enhancement of purification and antifouling performance. This transition can support the development of more efficient and sustainable membrane-based water-treatment technologies.

Future work should focus on four priorities: (i) shifting attention from interfacial deposition outcomes to the formation, heterogeneity, and evolution of bulk-phase aggregates; (ii) integrating mechanistic models, advanced characterization, and interpretable AI methods to improve predictive accuracy and model transferability; (iii) establishing quantitative structure–effect relationships between fouling-layer microstructure and emerging-contaminant rejection; and (iv) resolving the dynamic mismatch between membrane flux and catalytic oxidation rate in catalytic membrane systems. In particular, future AI applications should move beyond purely data-driven prediction toward physically informed and interpretable models. Standardized datasets that include water chemistry, aggregate descriptors, membrane properties, operating conditions, and fouling-layer characteristics are needed to improve model transferability. Combining AI with mechanistic models such as XDLVO theory, resistance-in-series analysis, and fouling kinetics may help overcome current limitations related to black-box prediction, data dependence, and poor extrapolation across membrane systems. Overall, research on organic membrane fouling is moving from phenomenological description toward mechanistic analysis and from single-purpose fouling mitigation toward the synergistic enhancement of purification and antifouling performance, which will support the development of efficient, green, and sustainable membrane-based water treatment technologies.

## Figures and Tables

**Table 1:** Quantitative comparison of organic aggregate properties and membrane-fouling parameters under different ionic conditions.

Foulant System and Ionic Condition	Membrane and Operation	Condensed Quantitative Findings	Main Implication	Ref.
Humic acid, 100 mg/L, pH 7.0; Ca^2+^ = 0, 1.5, and 5.0 mM	PVDF ultrafiltration; cross-flow; 2 bar; 0.1 μm; 140 kDa MWCO	Mean floc size increased from 3.31 to 76.04 and 107.28 μm. Postfiltration flux increased from 6.3% to 9.6% and 18.8% of the virgin-membrane flux, while cleaned-flux recovery increased from 41.1% to 59.1% and 79.5%.	Ca^2+^ bridging enlarged humic-acid flocs, reduced pore blocking, and improved hydraulic cleaning recovery.	[42]
Humic acid and colloidal silica; organic–inorganic mixed fouling	Ceramic ultrafiltration; cross-flow	The permeate-flux range decreased from 205–850 L m^−2^ h^−1^ for humic acid alone to 109–394 L m^−2^ h^−1^ in the presence of colloidal silica.	Colloidal silica intensified humic-acid fouling and altered the balance between reversible cake resistance and irreversible fouling.	[43]
Sodium alginate; increasing Ca^2+^ concentration	Membrane filtration of alginate gel	Specific filtration resistance exhibited a unimodal response to Ca^2+^ concentration, reaching a maximum of 3.63 ×10^15^ m kg^−1^.	Ca^2+^ did not aggravate alginate fouling monotonically; the most resistant gel formed within an intermediate Ca^2+^ range.	[44]
Alginate-based model extracellular polymeric substances; varied free Ca^2+^ concentration and ionic strength	Flat-sheet cross-flow filtration; flux-step tests	Increasing ionic strength reduced fouling by 66–72% under high-fouling conditions, whereas fouling reversibility decreased with increasing Ca^2+^ and reached approximately 3%.	Ca^2+^ promoted less reversible alginate fouling, whereas elevated ionic strength could suppress fouling by modifying Ca^2+^–carboxyl interactions.	[45]
Sodium alginate, 50 ppm; 0.125 mM Ca^2+^ or Mg^2+^	PES-MPC hollow-fiber membrane; outside-in filtration	In the Ca^2+^ system, relative permeability decreased by up to 82% after 60 min, and the maximum cake-layer thickness reached 7.59 × 10^−7^ m.	Ca^2+^ compacted the alginate cake layer and impaired hydraulic performance more strongly than Mg^2+^.	[46]
Alginate blocks, particularly MM blocks; Ca^2+^ = 0, 1, and 2 mM	Cross-flow ultrafiltration; 2 bar; 20 kDa membrane	Transparent exopolymer particle concentration increased from 4.0 to 9.6 and 17.1 mg Xeq L^−1^. Total organic carbon rejection reached 96.0% and 97.2% at 1 and 2 mM Ca^2+^, respectively.	Ca^2+^ promoted transparent exopolymer particle formation and accelerated cake-layer development.	[47]
Practical extracellular polymeric substances and model sodium alginate; varied Ca^2+^ addition order, concentration, dose volume, and dosing flow rate	Dead-end ultrafiltration; 10 kDa membrane; 20 kPa	Changing the Ca^2+^ dosing mode shifted the zeta potential from −7.47 to −5.99 mV for extracellular polymeric substances and from −19.1 to −15.1 mV for sodium alginate. Increasing the dosing flow rate from 5 to 25 mL/min shifted the zeta potential from −29.0 to −15.1 mV.	Aggregate properties and filtration resistance depended not only on Ca^2+^ concentration but also on addition order, dose volume, and dosing rate.	[48]

**Table 3:** Representative effects of fouling layers on emerging-contaminant removal in membrane processes.

Fouling Condition	Membrane Process and Target	Effect on Removal	Main Mechanism	Ref.
Organic fouling under different water-matrix conditions	Nanofiltration; antibiotics and estrogens	Fouling caused compound-dependent changes in rejection rather than a uniform decrease.	The effect depended on contaminant charge and hydrophobicity, membrane properties, and Ca^2+^-mediated interactions.	[56]
Combined organic fouling and inorganic scaling	Nanofiltration; pharmaceuticals and personal care products	Fouling and scaling reduced the retention of some trace organic contaminants despite the high intrinsic rejection of the membrane.	Cake-enhanced concentration polarization increased contaminant accumulation near the membrane surface and promoted transport across the membrane.	[57]
Organic fouling-layer formation	Nanofiltration; representative organic contaminants	Changes in rejection depended on both the membrane and contaminant properties.	The fouling layer altered membrane surface charge, hydrophobicity, effective pore structure, and local mass-transfer conditions.	[58]
Humic-acid/Ca^2+^ fouling	Nanofiltration; acetaminophen, sulfamethoxazole, and triclosan	Fouling and Ca^2+^ produced different rejection responses among the three contaminants.	Size exclusion, electrostatic interactions, and concentration polarization contributed simultaneously, with their relative importance depending on contaminant properties.	[59]
Alginate or humic-acid fouling during electrochemical filtration	Electroactive membrane; sulfamethoxazole	Alginate fouling enhanced sulfamethoxazole degradation, whereas humic-acid fouling inhibited it.	Alginate promoted adsorption and transport toward the reactive membrane surface, whereas humic substances hindered interfacial degradation through site shielding and competitive interactions.	[60]

**Table 4:** Representative quantitative outcomes of integrated water-purification and fouling-control strategies.

Strategy and Representative Conditions	Condensed Quantitative Outcome	Main Implication	Ref.
Trace homogeneous Fenton pretreatment before membrane filtration: 0.1 mM Fenton reagent, Fe^2+^:H_2_O_2_ = 1:1, neutral simulated wastewater.	During 2 h of filtration, the fouling rate decreased from 83% to 24%, and flux recovery increased from 44% to 98%. The pH decreased to 4.1 without external acid addition.	Low-dose Fenton pretreatment simultaneously suppressed fouling and improved fouling reversibility through in situ acidification and oxidation.	[69]
Fe(II)/peracetic acid pretreatment before ultrafiltration of secondary effluent: Fe(II) with 200 μM peracetic acid.	Total fouling resistance was reduced by 90.2%, compared with 29.7% for peracetic acid alone and 64.3% for Fe(VI)/peracetic acid.	Fe(II) activation provided the strongest fouling-control performance among the compared peracetic-acid pretreatments.	[70]
Fe^3+^ coagulation/co-catalytic membrane system: Fe^3+^ coagulation combined with a MoS_2_ membrane for peroxymonosulfate activation.	The pollutant degradation rate was 2.6-fold that of the catalytic membrane alone.	Fe^3+^ served as both a dissolved-organic-matter coagulant and a co-catalyst, coupling upstream foulant removal with interfacial pollutant degradation.	[74]

## Data Availability

No new data were created or analyzed in this study. Data sharing is not applicable to this article.
